# Integrative Study on Flow Characteristics and Impact of Non-Submerged Double Spur Dikes on the River System

**DOI:** 10.3390/ijerph20054262

**Published:** 2023-02-27

**Authors:** Zhenghua Gu, Xiaomeng Cao, Minxiong Cao, Weizhen Lu

**Affiliations:** 1 College of Civil Engineering and Architecture, Zhejiang University, Hangzhou 310058, China; 2 Key Laboratory of Port, Waterway & Sedimentation Engineering, Ministry of Communications, Nanjing 210029, China; 3 Teaching Center, Zhejiang Open University, Hangzhou 310012, China; 4 Department of Architecture and Civil Engineering, City University of Hong Kong, Hong Kong, China

**Keywords:** spur dike group, dike spacing, river system, CFD, flume experiment

## Abstract

The flow characteristics around non-submerged spur dikes continuously placed in the channel on the same side with orthogonal angle to the wall were investigated by numerical simulations and experimental measurements. Three-dimensional (3D) numerical simulations with the standard *k*−*ε* Model for incompressible viscous flow based on finite volume method and the rigid lid assumption for free surface treatment were conducted. A laboratory experiment was applied to verify the numerical simulation. The experimental data indicated that the developed mathematical model can effectively predict 3D flow around non-submerged double spur dikes (NDSDs). The flow structure and turbulent characteristics around them were analyzed and it was found that a distinct cumulative effect of turbulence exists between the dikes. By examining the interaction rules of NDSDs, the judgment criterion of spacing threshold was generalized as whether velocity distributions at the cross-sections of NDSDs along the main flow approximately coincided or not. It can be used to investigate the impact scale of the spur dike groups on the straight and prismatic channels and it is of great significance for artificial scientific river improvement and the assessment of river system health under human activities.

## 1. Introduction

The spur dike, one of the conventional hydraulic structures, is widely used to regulate the river system [1,2,3,4,5]. It can protect the riverside and riverbed from scouring and maintain the cross-section and original morphology of rivers [4,6,7]. Moreover, it can improve the ecological condition and enhance ecological diversity in the riverine environment [8,9]. With the existence of spur dikes, the hydrodynamic characteristics of the surrounding flow become complex and induce local scour or deposition in the vicinity. They also influence the positions and directions of mainstream and shore current, and further adjust the river way [10]. From a practical point of view, spur dikes exert influence on the river system in the form of groups [11,12,13]. These spur dikes or groups influence each other within a certain range of dike spacings, while the influence gradually weakens out of this range [14,15,16,17,18,19,20,21]. The spur dike groups in river systems can be classified into large- and small-scale in terms of the interaction strength [19,21]. The large-scale group consists of multiple dikes or small-scale groups, which are independent of each other, while the small-scale group is composed of multiple non-independent spur dikes, which influence each other significantly and play a role in the form of a whole unit [19,21]. For the small scale, the local influence on the river system is considered. However, for the large scale, we mainly consider its overall influence on the river system, which can help to explain the integrated response after the construction of spur dikes.

Most research on the spur dike group is focused on the small scale and the local response of the river system, mainly including the calculation of water surface curve [22], the flow status and scour patterns under different dike spacings [15,23], and the reasonable spacing of spur dikes [11,14,24]. The integrated response of the river system caused by anthropogenic influences has been a recent topic [25,26,27,28], which is on the influence scale of the spur dike group and the overall impact of the large-scale group acting on the river system [19,21]. Therefore, it is necessary to understand the mechanism between the action scale of the spur dike group and its flow characteristics, and put forward the judgment criteria for the spacing threshold of spur dike interaction. In the past, there was still a lack of in-depth and systematic research results in this area. The spacing threshold of two adjacent spur dikes is usually used as the reference value to define the scales. The two adjacent spur dikes are treated in large-scale if the dike spacing larger than the threshold; otherwise, they are considered small-scale [21]. Hence, the double spur dikes (DSDs) can be approximately adopted as the generalized model to investigate the transitional region between large- and small-scale [10,19,21].

Theoretical analysis and experimental measurement were the main investigation approaches applied to spur dike hydraulics in the early stages [15]. Computational Fluid Dynamics (CFD) is a cost-efficient means for spur dike research commensurate with the development of computer and numerical skills [10,12,29]. So far, one-, two- and three-dimensional numerical simulations of the flow around spur dikes have been achieved [2,12,16,19,30,31], covering the models of Reynolds Stress Model, *k*−*ε* Model, and Large Eddy Simulation. Among them, the three-dimensional (3D) simulation presents distinct, inherent 3D features and can provide more accurate, detailed flow characteristics [22,31] as long as these models can be verified by observed data [32].

In this study, the mathematical model for describing the flow around non-submerged double spur dikes (NDSDs) with ipsilateral and orthogonal layout was established based on the observed data from a flume experiment. We then investigated the flow structure and turbulent characteristics around them through the 3D numerical model. The interaction between NDSDs was examined, and the definition and judgment criterion of the spacing threshold were proposed for practical purposes.

## 2. Mathematical Model

### 2.1. Governing Equations

**Figure 1:**
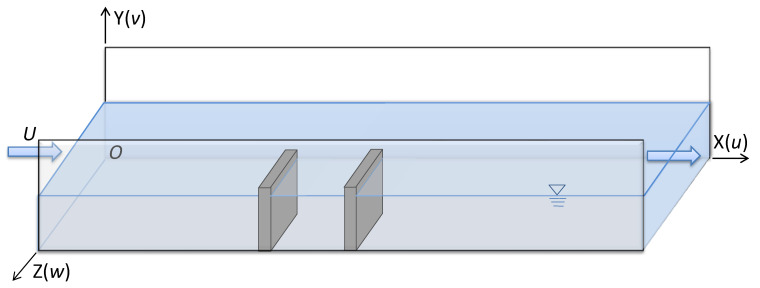
Ipsilateral and orthogonal NDSDs in flume.

The flow through NDSDs for this study is described in Figure 1. Two identical spur dikes are placed perpendicular to the shoreline and ipsilateral on the horizontal bed. The mean velocity of inflow is denoted *U*, and the coordinate is X along the flow direction, Y along water depth, and Z along the cross section. The governing equations of standard k−ε Model for incompressible viscous fluid can be expressed as [29]:

Continuity equation:

(1) $$\frac{\partial U_{i}}{\partial X_{i}}=0$$

Momentum equation:

(2) $$\frac{\partial U_{i}}{\partial t}+\frac{\partial }{\partial X_{j}}\left(U_{i}U_{j}\right)=-\frac{1}{\rho }\frac{\partial p}{\partial X_{i}}+\frac{\partial }{\partial X_{j}}\left[\left(\nu +\nu_{t}\right)\left(\frac{\partial U_{i}}{\partial X_{j}}+\frac{\partial U_{j}}{\partial X_{i}}\right)\right]$$

Turbulent kinetic energy equation:

(3) $$\frac{\partial k}{\partial t}+U_{i}\frac{\partial k}{\partial X_{i}}=\frac{\partial }{\partial X_{i}}\left[\left(\nu +\frac{\nu_{t}}{\sigma_{k}}\right)\frac{\partial k}{\partial X_{i}}\right]+G-\varepsilon$$

Turbulent dissipation rate equation:

(4) $$\frac{\partial \varepsilon }{\partial t}+U_{i}\frac{\partial \varepsilon }{\partial X_{i}}=\frac{\partial }{\partial X_{i}}\left[\left(\nu +\frac{\nu_{t}}{\sigma_{\varepsilon }}\right)\frac{\partial \varepsilon }{\partial X_{i}}\right]+C_{1\varepsilon }\frac{\varepsilon }{k}G-C_{2\varepsilon }\frac{\varepsilon^{2}}{k}$$

where *k* is the turbulent kinetic energy; ε is the turbulent dissipation rate. *U_i_* is the velocity component in three directions, i.e., *u*, *v*, *w*; ρ is the fluid density; *t* is the time; *p* is the pressure; ν is the coefficient of kinematic viscosity; $\nu_{t}$ is the eddy viscosity, and $\nu_{t}=\rho C_{\mu }k^{2}/\varepsilon$; *G* is the generating term $G=\nu_{t}\left(\frac{\partial U_{i}}{\partial X_{j}}+\frac{\partial U_{j}}{\partial X_{i}}\right)\frac{\partial U_{i}}{\partial X_{j}}$. The constants in the equations are as follows: $C_{1\varepsilon }=1.44$, $C_{2\varepsilon }=1.92$, $\sigma_{k}=1.0$, $\sigma_{\varepsilon }=1.3$, and $C_{\mu }=0.09$ [32]. Removing all transient terms in the above equations leads to the basic equations for the steady flow around spur dikes.

### 2.2. Grids and Boundary Conditions

The numerical model of the flow around NDSDs is built through the commercial CFD software FLUENT. The simulation area is divided into several regular blocks for generating meshes by adding some appropriate auxiliary surfaces. The meshes adopted are hexahedral structured grids refined in the vicinity of two spur dikes [19]. The pressure-based solver and the standard k−ε Model are selected. The inlet turbulence parameters of hydraulic diameter *D*_H_ and turbulence intensity *I* are calculated as below [32,33]:

(5) $$D_{H}=\frac{2A}{C}$$

(6) I=0.16ReDH−1/8

where A is the area of flow cross-section; C is the wetted perimeter; ReDH is the Reynolds number based on the hydraulic diameter $D_{H}$. The Pressure-Implicit with Splitting of Operators (PISO) algorithm is conducted for the pressure-velocity coupling; the Body Force Weighted (BFW) method is used for pressure discretization; and the discrete format of momentum, turbulent kinetic energy, and turbulent dissipation rate are all assumed with the first order upwind scheme. The inflow uses velocity-inlet as the upstream boundary condition. Because the water surface slope of non-submerged spur dike flow hardly changes in flat-bottomed flume tests, the rigid lid assumption is used to model the free surface [16], which assumes the free surface is constant. The top surface of water body uses symmetry, in which the tangential velocity may not be zero compared with the routine wall boundary. The flow at the downstream outlet is assumed as free outflow. The dike bodies and other faces of the flume are regarded as wall and meet the no-slip condition, and the standard wall functions are used to solve *k*, *ε* values near the wall.

### 2.3. Verification

**Figure 2:**
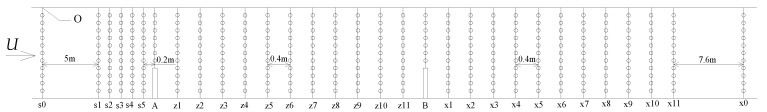
Distribution of measured cross-sections and points.

Verification data obtained from the flume experiment were collected with the multifunction flume of 50 m long, 1.2 m wide, and 1.4 m high at Jiangong Test Hall of Zhejiang University, China [19,21]. The spur dikes were made of plexiglass into 1.6 cm thick, 40 cm high, and 30 cm long. The water level was controlled at 0.3 m, and the flow rate of inflow was 0.058 m^3^/s to generate subcritical flow (the supercritical flow around spur dike may result in the break wave and not considered here). Nortec Vectrino Acoustic Doppler Velocimeters (ADVs) were used to measure velocities, and wave height meters for water surface elevations [19,21]. The distribution of measured cross-sections and points are shown in Figure 2. Initial cross-section s0 is at the inlet. Spur dike 1 (SD1) is at cross-section A, 6 m from the upstream inlet (20 times the length of spur dike); spur dike 2 (SD2) at cross-section B, 12 m from the downstream outlet (40 times the length of spur dike). Such a distance can eliminate the influence of the boundary on the results. There are 5 cross-sections (s1–s5) at the upstream of SD1 with equal spacing of 0.2 m; 11 cross-sections (z1–z11) between SD1 and SD2 with equal spacing of 0.4 m; and then 11 cross-sections (x1–x11) along the downstream of SD2 with equal spacing of 0.4 m. The outlet cross-section x0 is 7.6 m from cross-section x11. The total numbers of measured cross-sections and points are 31 and 341, respectively. The coordinate origin is arranged at the bottom of flume at the point *O* in Figure 2. With the reference of the flume experiments, the geometry dimension for numerical model is 22.8 m long, 0.3 m high, and 1.2 m wide, in which the total grid number is about 150,000 under the mesh size of ΔX = 0.20 m, ΔY = 0.04 m, ΔZ = 0.05 m, and grid refined within 1 m around the spur dike.

**Figure 3:**
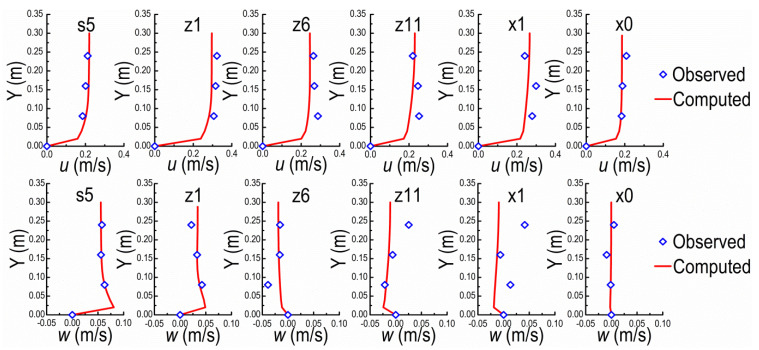
Distributions of *u*, *w* along Y at verification cross-sections on the central axis of flume.

**Figure 4:**
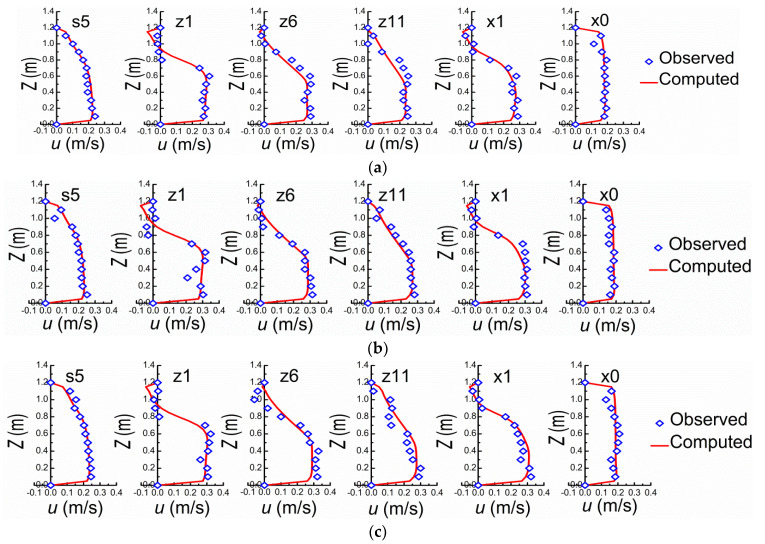
Comparisons of *u* along Z between observed and computed. (**a**) Y = 0.08 m. (**b**) Y = 0.16 m. (**c**) Y = 0.24 m.

Sections of s5, z1, z6, z11, x1, and x0 are selected to verify the mathematical model (Figure 2). The mean velocities at inlet cross-section in X, Y, and Z directions are respectively set according to the observed data from flume experiment. It should be pointed out that all velocities in this study are time-averaged velocities except the fluctuating ones used for estimating the turbulence intensity σ herein. The comparisons between the observed and computed results are shown in Figure 3 and Figure 4, in which the verifications of velocities (*v*) are ignored due to the small magnitudes comparing to *u* and *w*. For *u* and *w* along Y on the central axis of flume at different verification cross-sections, the comparisons between observed and computed are shown in Figure 3. The comparisons of *u* along Z direction between observed and computed values on different horizontal planes (Y = 0.08, 0.16, and 0.24 m) are shown in Figure 4. In general, the experimental and computational data agree well. It should be pointed out that the measured data may themselves be subject to measurement error and expand on what these might be, i.e., it cannot be guaranteed that each and every measurement is accurate. The numerical model can be used for subsequent investigation on the structure characteristics of the flow field around NDSDs and their interaction laws. The verification performance of *w*, however, is relatively worse than *u*, because of the comparatively small magnitude. 

## 3. Results and Discussions

### 3.1. Flow Structure

**Figure 5:**
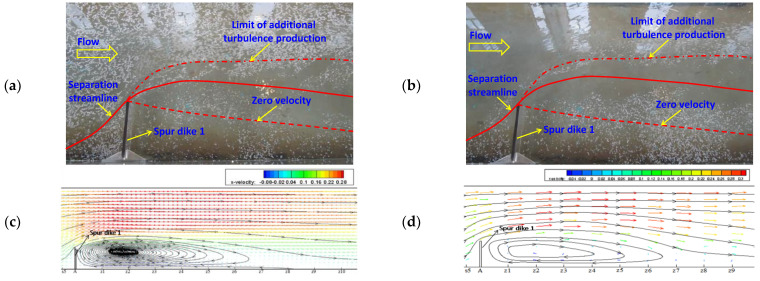
The flow structure near spur dike 1: (**a**) surface image in initial stage of visualization, (**b**) surface image in last stage of visualization, (**c**) numerical result on the horizontal plane of Y = 0.08 m, and (**d**) experimental result on the horizontal plane of Y = 0.08 m.

**Figure 6:**
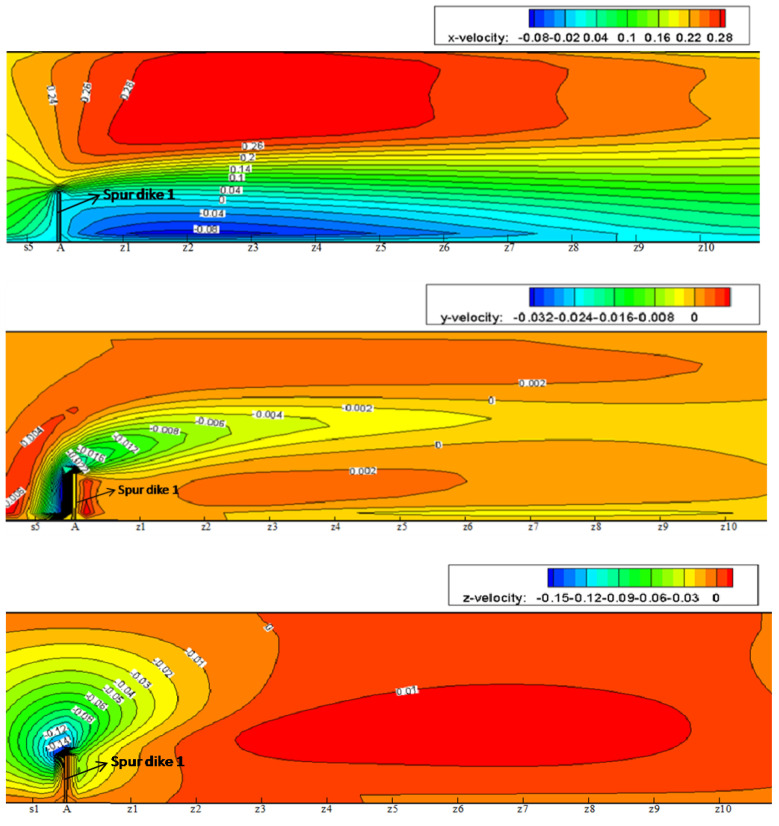
Velocity contours in X-, Y-, and Z-direction on the horizontal plane of Y = 0.08 m.

The flow structure can usually be classified into three types, i.e., micro-flow, meso-flow, and macro-flow according to hydraulic research scale. Concerning the micro-flow, attention is paid to the water movement at molecule level; and for the meso-flow structure, we are more interested in flow characteristics of turbulence; while dealing with the macro-flow, more attention is focused on the flow process in natural scale and the mean flow parameters in time series. In this study, the flow type belongs to the macro-flow structure. The simulation results of the verification condition are subsequently used to analyze the flow patterns of NDSDs. Figure 5a,b respectively show the surface patterns in the initial and the last stages of the visualizing test. A flow separation zone around the spur dike similar to the result of Ettema and Muste [34] could be observed. Velocity vectors and stream lines are presented to compare experimental and numerical results (see Figure 5c,d). Due to the water-blocking, deflecting, and narrowing effects of the spur dike, the flow velocities decreased and the flow developed into the backflow zone at the upper corner of the spur dike; on the other hand, the mainstream was detoured and deflected toward the other side of flume with the increase of flow velocities as well as the deflection of mainstream extending for a long distance. Meanwhile, the downstream backflow zone appeared at the lower corner of the spur dike because of flow separation. After surpassing the downstream backflow zone, the mainstream entered recovery zone and was gradually restored. Figure 6 presents the velocity contours in X, Y, and Z directions near SD1. The contour profile of *u* is similar to the streamline distribution. The negative velocity curves of *v* from the upper corner of spur dike extend downstream along the dike tip, and restore gradually, which stretched in a shuttle shape. Taking the dike tip as source point, the negative velocity distribution of *w* radiates outward and decreases gradually. In terms of the velocity restoring process, it presents the slowest in the X-direction but fastest in the Z-direction which indicates that the velocity *u* plays a dominant role to form the flow pattern of NDSDs, while the *v* and *w* affect the shape and internal structure of the backflow zone.

**Figure 7:**
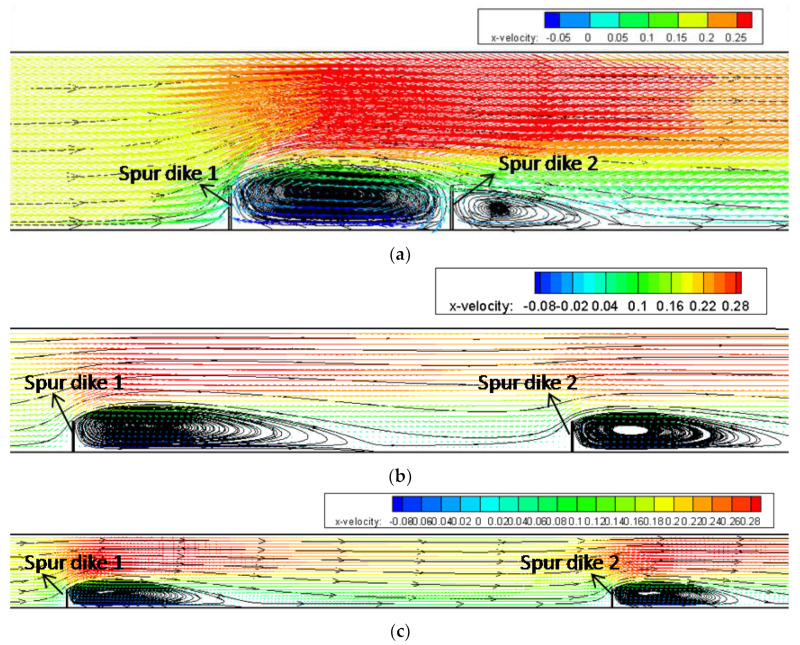
The velocity fields on the horizontal plane of Y = 0.08 m under different dike spacings. (**a**) *s* = 1.5 m (5*b*). (**b**) *s* = 4.8 m (16*b*). (**c**) *s* = 9 m (30*b*).

The cases with different spacings (*s*) between two dikes were conducted in the numerical model to estimate the effect of *s*. We set three different spacings, i.e., 1.5 m (5*b*), 4.8 m (16*b*), and 9 m (30*b*), where *b* is the length of spur dike. The increasing of *s* could decrease the interaction between dikes (shown Figure 7). They strongly influence each other at *s* = 1.5 m, and the length of the downstream backflow zone at SD1 is close to the spacing (*s*), but the length and the width of the downstream backflow zone at SD2 decrease significantly. For *s* = 4.8 m, the length of the downstream backflow zone at SD1 is less than the dike spacing, and the influence of SD1 acting on SD2 decreases. As for *s* = 9 m, the dike spacing is much larger than the length of the downstream backflow zone at SD1. The recovery zone between dikes starts and the flow patterns near the two spur dikes are very similar, which indicate that the interaction between them has disappeared in general. More discussions will be presented in Section 3.3.

### 3.2. Turbulent Characteristics

**Figure 8:**
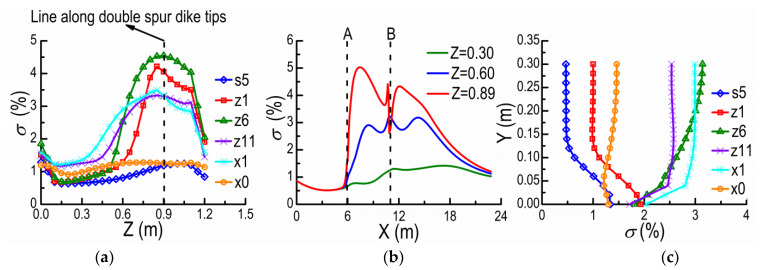
Turbulence intensities along different directions. (**a**) Along Z-direction (Y = 0.08 m). (**b**) Along X-direction (Y = 0.08 m). (**c**) Along Y-direction (Z = 0.6 m).

The spur dike turbulence intensity is estimated and shown in Figure 8. It belongs to medium intensity turbulence within 1–5% [33]. Figure 8a shows the distribution of the turbulence intensity σ along Z-direction, on the horizontal plane of Y = 0.08 m, at s5, z1, z6, z11, x1, and x0, where *σ* is defined as the ratio of the root-mean-square of the fluctuating velocity to the time-averaged velocity [33]. The turbulent intensities are very small at the upstream cross-section s5 and the downstream outlet x0, and are almost unchanged along the Z-direction. However, the turbulent intensities increase with their positions approaching the line along double spur dike (DSD) tips at the cross-sections between dikes (z1, z6, z11) and the downstream x1 of SD2. They all reach the maximum at the dike tips (Z = 0.9 m). The variation range of turbulent intensity at the cross-section z6 approaches the largest, and the maximal turbulent intensity approximately occurs here. Figure 8b reveals the variations of σ along X-direction, on the horizontal plane of Y = 0.08 m, at Z = 0.3, 0.6, 0.89 m. As shown in Figure 8b, the obviously cumulative phenomenon exists on the turbulent intensity curves in the line along DSD tips and its vicinity. The above results indicate that: (i) the spur dike tips present strong disturbance on the flow; (ii) the turbulent intensity reaches the maximum at the downstream of spur dike, then gradually restores; (iii) for the flow around spur dike, its fluctuating characteristics is more difficult to recover than its time-averaged velocity field; (iv) there is a cumulative effect of flow fluctuating between NDSDs. Figure 8c shows the changes of the turbulent intensity σ along Y-direction, on the central-axis plane of Z = 0.6 m at s5, z1, z6, z11, x1, and x0. There is a significant difference among the distributions of the turbulent intensity with water depth, at different cross-sections. The turbulent intensities at the cross-sections near SD1 (s5 and z1) are small and gradually increase downwards from the water surface. The turbulent intensities near SD2 (z11 and x1) and between DSDs (z6) are large and gradually decrease downwards from the water surface. The turbulent intensities at x0, however, are almost unchanged along Y-direction. These also illustrate the cumulative effect of flow fluctuating and the self-adjusting action of flow between DSDs.

### 3.3. Interaction between Double Spur Dikes (DSDs)

#### 3.3.1. Lower Spur Dike (SD2) Acting on Upper Spur Dike (SD1)

**Figure 9:**
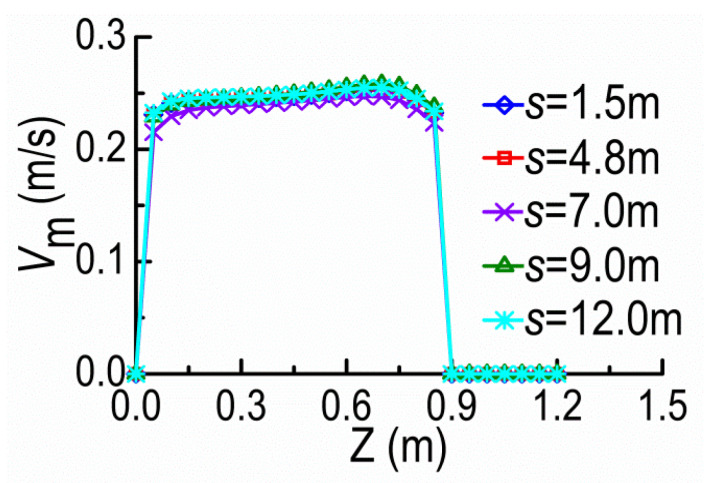
*V*_m_ along Z-direction at the cross-section A.

**Figure 10:**
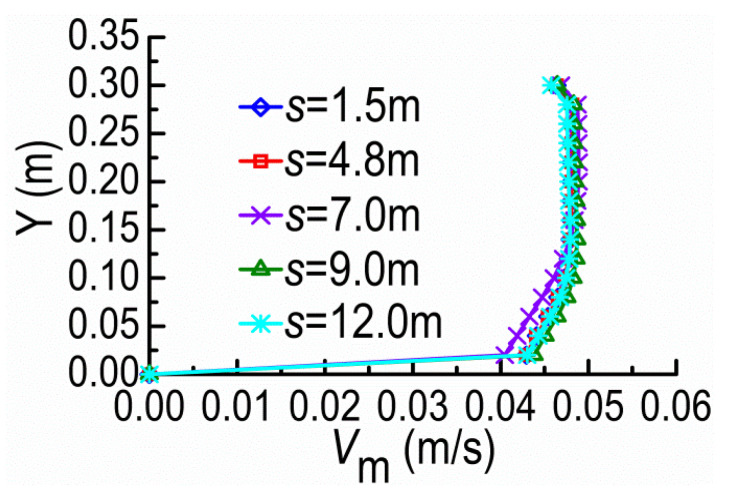
*V*_m_ along Y-direction at the tip vicinity of SD1.

Figure 9 and Figure 10 show the velocity magnitude *V*_m_ distributions along the Z-direction at the cross-section A and the Y-direction at the tip vicinity of SD1, on the horizontal plane of Y = 0.08 m, under different dike spacings (*s*). The velocities at the cross-section A barely change for small *s* because SD2 remains in the shield area of SD1 where the mainstream is still constrained by SD1 and the mainstream is kept outside DSDs. With the spacing over the certain extent, the mainstream begins to diffuse and restore; meanwhile, SD2 acting on SD1 gradually fades with the former moving downstream.

#### 3.3.2. Upper Spur Dike (SD1) Acting on Lower Spur Dike (SD2)

**Figure 11:**
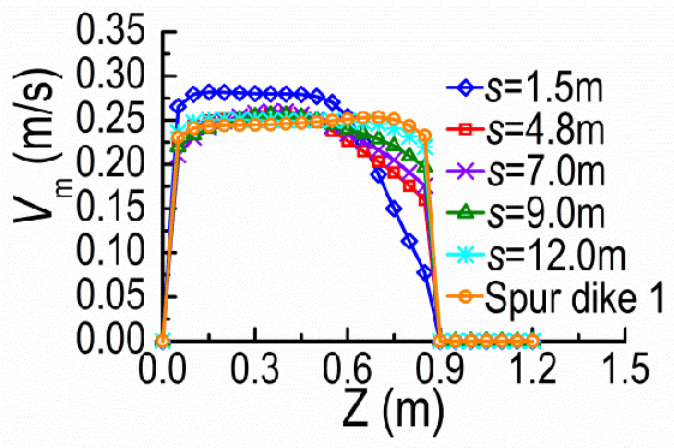
*V*_m_ along Z-direction at the cross-section B.

**Figure 12:**
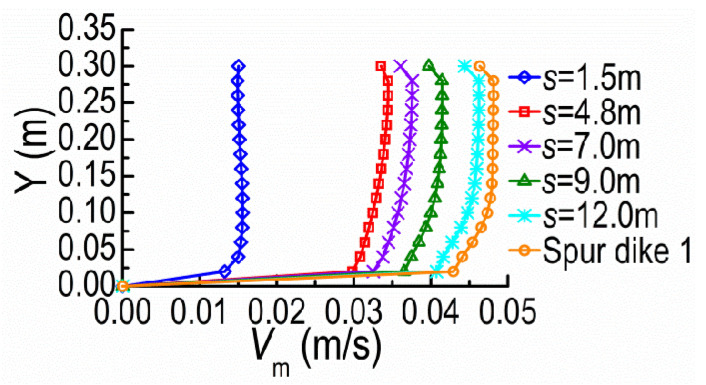
*V*_m_ along Y-direction at the tip vicinity of SD2.

Figure 11 and Figure 12 present the velocity profiles along the Z-direction at the cross-section B and the Y-direction at the tip vicinity of SD2, on the horizontal plane of Y = 0.08 m, under different dike spacings. With increasing *s*, the distribution of velocities along Z-direction at the cross-section B becomes uniform and the maximum *V*_m_ decreases (see Figure 11). The velocities at the tip vicinity of SD2 increase with the *s* increasing (see Figure 12). The vertical velocity distributions approach the distribution pattern at the tip vicinity of SD1. This is for **a** similar reason that SD2 lies in the shield area of SD1 for the small *s* and the mainstream concentrates outside DSDs, which results in the small velocity magnitudes at the tip vicinity of SD2. Moreover, the mainstream begins to diffuse and the velocities at the tip vicinity of SD2 gradually increase with SD2 moving downstream to certain extent. The interactions between DSDs become very weak and the velocity distribution at the cross-section B is close to A, if the recovery zone of mainstream occurs between DSDs.

#### 3.3.3. Discussion on the Spacing Threshold of Double Spur Dikes (DSDs)

The research on the layout spacing of spur dikes was mainly focused on how to choose reasonable dike spacing to achieve the best training effect from the points of river regulation and riverbank protection, and the empirical dike spacing was generally considered to be 2–4 times of spur dike length [14]. The local response of the river system was the main interest and the combination of spur dikes was attributed to the spur dike group in the small scale. However, to investigate the integrated influence of spur dike group acting on river system, the spur dikes on river system are necessarily clustered into the spur dike groups in a large or small scale. In this study, the transition of the interaction between NDSDs was discussed. From Section 3.2, we conclude that the interaction can be neglected only for the very large dike spacing because the turbulent characteristics of the spur dike restores more difficultly than macro-flow structure does. Concerning mainly the macro-flow structure for investigating the integrated response of the river system, we can simply adopt the judgment criterion based on the restoration of macro-flow structure instead of the restoration of turbulent characteristics while investigating the point of transition of DSDs interaction. We adopted whether the lateral distributions of velocities at adjacent two spur dikes are similar or not, and the corresponding dike spacing is defined as the spacing threshold of NDSDs with ipsilateral and orthogonal layout in straight prism channel [21]. According to the analysis in Section 3.1, we know that the spacing threshold in this definition is obviously greater than the sum of the lengths of downstream backflow zone at SD1 and the upstream backflow zone at SD2.

**Figure 13:**
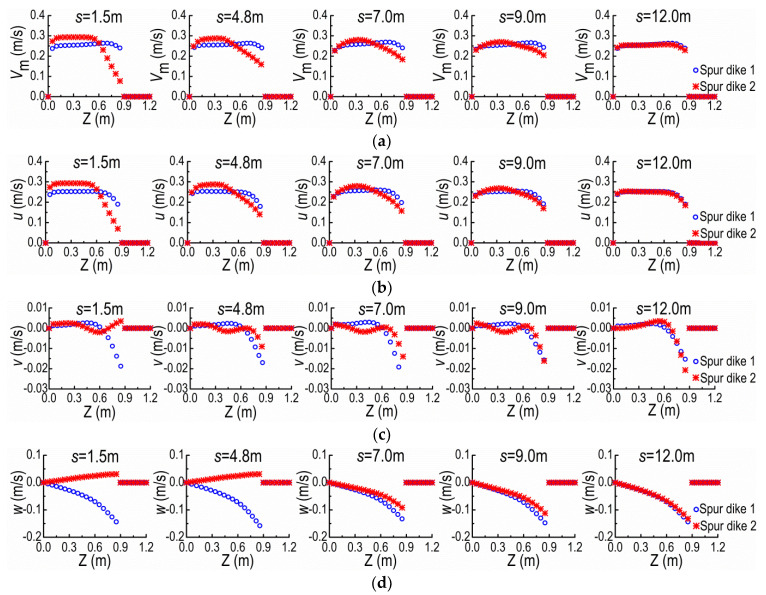
Velocity distributions along Z-direction at SD1 and SD2 under different *s* values. (**a**) *V*_m_ curves. (**b**) *u* curves. (**c**) *v* curves. (**d**) *w* curves.

With moving SD2 away from SD1 under the same flow condition, Figure 13 shows the distributions of *V*_m_, *u*, *v* and *w* at the cross-sections A and B at Y = 0.15 m for the *s* = 1.5 m (5*b*), 4.8 m (16*b*), 7.0 m (23.3*b*), 9.0 m (30*b*) and 12.0 m (40*b*). The distribution curves of velocity become more and more similar at the cross-sections A and B as increasing *s*. For *s* = 12 m, both curves are approximately the same, and this scale is close to the spacing threshold of NDSDs under the same flow condition. Furthermore, when the distributions of *V*_m_ are mostly coincident at the cross-sections A and B, the curves of *u*, *v*, and *w* at the same locations are also coincident, and the profiles of *u* and *V*_m_ are similar. Combining the previous results in Section 3.1, we can deduce that the similarity of *u* at the cross-sections of adjacent upper and lower spur dikes should be used as the criterion of the spacing threshold. In addition, we can estimate whether the velocity distributions at the cross-sections of adjacent two spur dikes are coincident or not according to the coincidence of velocities at their tips (Figure 13). To determine the spacing threshold conveniently, we can use the average velocity distributions along the water depth to replace the velocity distributions in these judgment criteria.

## 4. Conclusions

Comparing numerical simulation and the flume experiment, the verification results indicate that the developed model can produce accurate simulation on the 3D flow around NDSDs in the flat flume. Moreover, the model can be easily built to obtain the water flow characteristics around dikes. Through this model, we can profoundly explore the flow structure and turbulent characteristics of NDSDs and the laws of their interaction.

Compared to the velocities in Y- and Z-directions, the velocities in X-direction play a dominant role to affect the macro-flow structure of spur dikes, while the velocities in Y- and Z-directions have an important influence on the shape and internal structure of backflow zones. Moreover, the velocities in X-direction of the spur dike flow are the slowest to restore, followed by the velocities in Y-direction, and the fastest in Z-direction. We also notice that the tips of spur dikes strongly disturb the flow. The turbulent intensity reaches the maximum at the downstream vicinity of spur dike, then gradually restores; the fluctuating characteristics of the flow around the spur dike are more difficult to restore than its time-averaged velocity field; and the cumulative effect of flow fluctuating occurs between NDSDs.

For different dike spacings, the velocities at the cross-section of upper spur dike are almost unchanged, while the distributions of *V*_m_ at the cross-section of lower spur dike change greatly. When the dike spacing increases to the recovery zone of mainstream occurring between DSDs, the interaction between the two spur dikes has been very weak and the velocity distribution at the cross-section of lower spur dike is close to that at the cross-section of upper spur dike. For the non-submerged spur dikes with ipsilateral and orthogonal layout and same length in the straight prism channel, we can utilize the similarity of *u* at the cross-sections of adjacent two spur dikes as the criterion of the spacing threshold. These conclusions are useful for investigating the influence scale of multiple spur dikes, and the hydrodynamic process of spur dike group acting on river system. As for practical purposes, these achievements can be applied to scientifically restore rivers and correctly assess the health of river systems affected by human activities. The future works related to this research will focus on the interaction effects of the multiple spur dikes under different dike types, layouts, and flow conditions to obtain the empirical formulas of the spacing thresholds.

## Data Availability

Not applicable.
